# Ultra-high Photoresponsivity in Suspended Metal-Semiconductor-Metal Mesoscopic Multilayer MoS_2_ Broadband Detector from UV-to-IR with Low Schottky Barrier Contacts

**DOI:** 10.1038/s41598-018-19367-1

**Published:** 2018-01-19

**Authors:** Gustavo A. Saenz, Goran Karapetrov, James Curtis, Anupama B. Kaul

**Affiliations:** 1 Department of Materials Science and Engineering, PACCAR Technology Institute, The University of North Texas, Denton, TX 76203 USA; 2 Department of Electrical Engineering, The University of North Texas, Denton, TX 76203 USA; 3 Department of Electrical and Computer Engineering, The University of Texas at El Paso, El Paso, TX 79968 USA; 4 Department of Physics, Drexel University, Philadelphia, PA 19104 USA

## Abstract

The design, fabrication, and characterization of ultra-high responsivity photodetectors based on mesoscopic multilayer MoS_2_ is presented, which is a less explored system compared to direct band gap monolayer MoS_2_ that has received increasing attention in recent years. The device architecture is comprised of a metal-semiconductor-metal (MSM) photodetector, where Mo was used as the contact metal to suspended MoS_2_ membranes. The photoresponsivity ${\boldsymbol{R}}$ was measured to be ~1.4 × 10^4^ A/W, which is > 10^4^ times higher compared to prior reports, while the detectivity D* was computed to be ~2.3 × 10^11^ Jones at 300 K at an optical power *P* of ~14.5 pW and wavelength *λ* of ~700 nm. In addition, the dominant photocurrent mechanism was determined to be the photoconductive effect (PCE), while a contribution from the photogating effect was also noted from trap-states that yielded a wide spectral photoresponse from UV-to-IR (400 nm to 1100 nm) with an external quantum efficiency (EQE) ~10^4^. From time-resolved photocurrent measurements, a decay time *τ*_*d*_ ~ 2.5 ms at 300 K was measured from the falling edge of the photogenerated waveform after irradiating the device with a stream of incoming ON/OFF white light pulses.

## Introduction

Two-dimensional (2D) layered materials have inspired multiple research platforms in recent years due to their extraordinary mechanical, electrical, and optoelectrical properties^1,2^. In addition to their intriguing materials properties, some exciting applications of 2D materials include their use in high-sensitivity strain sensors^3^, flexible electronics enabled by additive manufacturing techniques^4^, electronic devices exhibiting interesting excitonic effects^5^, and heterostructure-based biomedical devices for implantable electronics^6^. A family of two-dimensional materials, the transition metal dichalcogenides (TMDs), such as MoS_2_ have long been used as a lubricant in numerous technological applications such as space-based components and systems, as well as in industry^7^. Renewed interest in these van der Waals solids has emerged in recent years with our ability to isolate mono-, few-layer, and multilayer (ML) atomically thin nanomembranes that provide a platform for unveiling their intriguing electronic and optoelectronic properties^1^. In particular, since the advent of graphene, MoS_2_ is one of the most widely studied TMDs with device demonstrations that have already yielded high-performance transistors based on 6.5 Å thick monolayer membranes, where the MoS_2_ typically displays *n*-type conductivity^8^. The 2H-MoS_2_ crystal structure is the most stable polytype found in nature and contains layers centrosymmetric to each other, creating a hexagonal motif. An important characteristic of MoS_2_ is that the band gap changes from indirect in the bulk (at ~1.2 eV between the *Γ* and *K*-points) to direct gap in single layers (~1.8 eV at the *K*-point in the first Brillouin zone)^9^. Moreover, the band gap can be tuned by the application of an external electric field^10^ or via mechanical strain^11^. It comes as no surprise then, that this material has tremendous potential for next generation electronic and optoelectronic applications.

In particular, photodetection in MoS_2_, just as in some other TMDs and elemental black phosphorus^12^, can be engineered to be wavelength-selective by varying the layer-number^13^. In addition, the high photoresponsivity in the visible range of the spectrum for MoS_2_ comes from the presence of van Hove singularities in the electronic density of states near the band edge^14^. On the other hand, monolayer MoS_2_ is shown to absorb ~10% of the incoming light with a power conversion efficiency of 1%^15^. Moreover, it has been demonstrated that the photocurrent in MoS_2_ is not only generated by the commonly observed photoconductive effect, but there is also a contribution from the photothermoelectric effect below band gap illumination which is attributed to the high Seebeck coefficient mismatch at the metal-semiconductor junction, as well as the photovoltaic effect^16^. In addition, ML MoS_2_ is not widely studied, despite the broader spectra responsivity spanning the ultraviolet (UV) to the near infrared (NIR)^17^. These indicators of strong light-matter interactions make MoS_2_ especially attractive for photodetector PD applications. In this work, a mesoscopic ML suspended MoS_2_ PD has been designed and characterized, where the responsivity ${\boldsymbol{R}}$ was measured to be ~1.4 × 10^4^ A/W and the detectivity D* was determined to be ~2.3 × 10^11^ Jones for a wavelength *λ* of 700 nm. These performance metrics are unparalleled and amongst the highest reported to our knowledge for few-layer MoS_2_ photodetectors, and far surpass ML devices^17–21^. However, more complex structures have been explored to improve the MoS_2_ PDs performance by using several techniques such as surface plasmons^22^, integrated waveguides^15^, quantum dots^23^, and heterostructures^24–26^.

Here, besides the photodetector device measurements, we perform a multifaceted investigation comprising of atomic force microscopy (AFM), photoluminescence (PL) and Raman spectroscopy to analyze the structural and chemical characteristics to help shed insights on the origins of the superior optoelectronic device performance. The nanofabrication process is described in detail which resulted in low Schottky barrier contacts, one of the key factors in yielding the high device performance metrics we observe here. The optoelectronic device measurements were conducted in vacuum using a cryogenic probe stage equipped with a cryostat, where measurements were conducted from ~5.4 K to 350 K. The combined techniques allowed us to conduct a comprehensive analysis to empirically compute the device parameters, such as the Schottky barrier height *φ*_*SB*_, field effect mobility *μ*_FE_, ${\mathcal R}$, D*, external quantum efficiency EQE, and time decay *τ*_*d*_, which were then compared to prior work. This work unequivocally pushes the state-of-the-art for yielding ultra-high performance MoS_2_ photodetectors that should pave the way for future optoelectronics applications arising from ML mesoscopic MoS_2_.

## MoS_2_ Device Architecture

**Figure 1:**
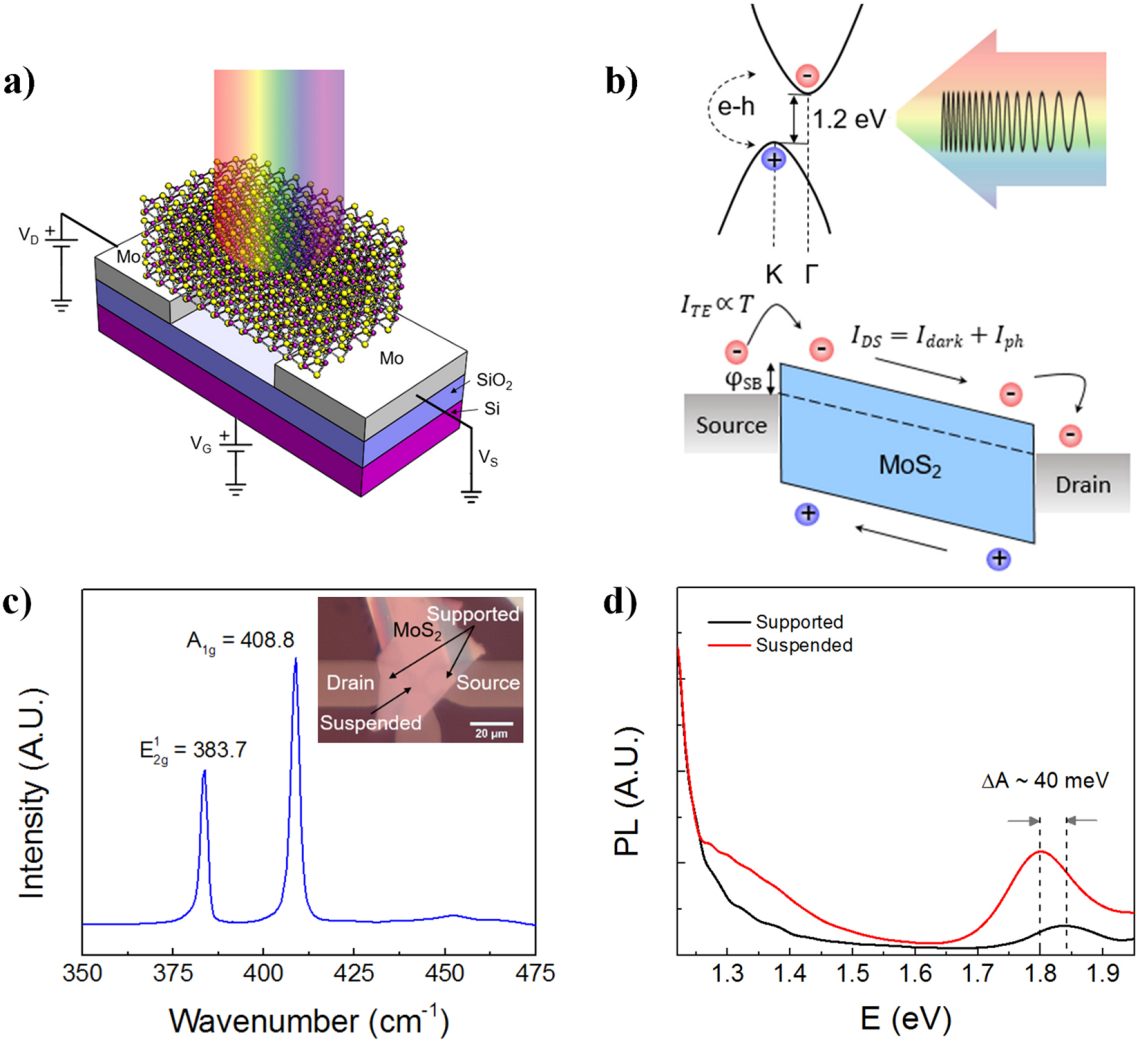
Schematic of the suspended ML MoS_2_ PD used in this work. (**a**) Two-terminal configuration of our device fabricated with 100 nm Mo bottom contacts formed on thermally oxidized (thickness of SiO_2_ ~ 270 nm) Si substrates. For three-terminal measurements that are reported later in the “Three-terminal Gating Measurements” Section, the substrate acts as the gate, where the gate voltage is shown as *V*_*G*_. (**b**) Top: The photocurrent generation mechanism is attributed to the excitation of e-h pairs from the valence band maximum at the *K*-point in the Brillouin zone, to the gamma *Γ*-point in the conduction band minimum which is offset in k-space relative to the *K*-point. Bottom: Energy band diagram of the PD under an applied source-drain bias voltage, where *I*_*ph*_ = *I*_*DS*_ − *I*_*dark*_. As temperature *T* increases, according to the thermionic emission model, the thermionic emission current *I*_*TE*_ increases since the carriers have more energy to overcome the Schottky barrier φ_SB_ at the interface. (**c**) Raman spectra showing the bulk MoS_2_ strong vibrational peaks ${E}_{{\rm{2g}}}^{1}$ and *A*_*1g*_ at 383.7 cm-1 and 408.8 cm^−1^, respectively. The inset shows the optical image of the MSM MoS2 PD. (**d**) PL of the suspended area compared to the supported regions, where a shift to the left of ~40 meV is observed in the *A1* peak attributed to direct hot-luminescence effects

The device schematic is outlined in Fig. 1a, where MoS_2_ is shown in its ML configuration, and pre-patterned sputtered Mo-contacts are used to contact the nanomembranes underneath to suspend the MoS_2._ Our architecture corresponds to a MSM configuration based on a suspended mesoscopic ML MoS_2_ diaphragm fabricated using an all-dry, ultra-clean stamping process. This process is described here and also discussed in more detail in the Supplementary Information Section. Invoking the photoconductive effect, where the incoming radiation has higher energy than the electronic band gap of the material, excites carriers from the valence band to the conduction band. This generates electron (*e*)-hole (*h*) pairs, as shown by the schematic at the top of Fig. 1b. As observed here, the valence band maximum at the *K*-point of the Brillouin zone, is offset relative to the minimum of the conduction band at the *Γ*-point, which illustrates the indirect nature of the optical excitation. After the photocarriers are generated through the absorption of incoming radiation, the *e*-*h* collection process at the respective electrodes determines the total photocurrent *I*_*ph*_ generated in the external circuit that is, to some degree, influenced by the nature of the semiconductor-to-metal contacts. The schematic in the bottom right of Fig. 1b shows the interface effects at the contacts, where metal contacts to semiconductors display a Schottky character as is often the case, though engineering Ohmic behavior is desirable for many high-performance electronic devices. However, it is difficult to form Ohmic contacts between a metal and a semiconductor, since the work function of most metals *φ*_*M*_ is larger than the electron affinity *χ* of the intrinsic semiconductor.

In reality, the Schottky barrier height *φ*_*SB*_ is ≫ 0 typically, and in order to maximize *I*_*ph*_, quantum efficiency and R, a *φ*_*SB*_ as low as possible is desired (see Fig. 1c). Several materials have been proposed to yield a low *φ*_*SB*_ with MoS_2_ given its low *χ*. Metals such as Au (*φ*_*SB*_ *=* 126 meV)^27^ and Ti (*φ*_*SB*_ *=* 65 meV)^28^ have been most commonly used to contact TMDs including MoS_2,_ and an ultra-thin Ti layer is often used as an adhesion promoter to the Au film on oxidized Si substrates. More exotic materials such as scandium (*φ*_*SB*_ = 30 meV)^29^ and alloys such as Permalloy Py (*φ*_*SB*_ = −5.7 meV)^30^ have also been utilized, resulting in a gate-tunable *φ*_*SB*_. Recently, using density functional theory (DFT) calculations, Kang *et al*.^31^ proposed the use of molybdenum Mo (*φ*_*Mo*_ ~ 4.5 eV) as a contact material to MoS_2_ and estimated the Schottky barrier height to be ~100 meV. Here, we experimentally validate the theoretical predictions by Kang *et al*.^31^ and, in fact, show the *φ*_*SB*_ to be lower by four times in our Mo-MoS_2_ contacted devices. Thus, we demonstrate Mo as an ideal contact metal to significantly reduce *φ*_*SB*_ which in turn enhances the photodetection performance metrics of our MoS_2_ devices.

Figure 1c displays the MoS_2_ Raman spectra where the supported and suspended regions of our device are shown in the inset. The spectra in Fig. 1c shows the strong MoS_2_ vibrational peak occurring at 383.7 cm^−1^ representing the ${E}_{{\rm{2g}}}^{1}$ mode while the *A*_*1g*_ mode occurs at 408.8 cm^−1^. In order to validate that the MoS_2_ diaphragm is indeed suspended, we rely on PL measurements where the PL spectra of the suspended and the supported regions are shown in Fig. 1d for the device in the inset of Fig. 1c. In Fig. 1d, the direct transition *A1* peak exhibits a red shift by ~40 meV for the suspended MoS_2_ in comparison to the supported MoS_2_ which is consistent with the findings of Scheuschner *et al*. where the red shift was measured to be 15 meV in the *A1* peak in bilayer MoS_2_^32^.

The device fabrication process involves alignment and transfer of mechanically exfoliated MoS_2_ nanomembranes onto pre-patterned Mo contacts resulting in a suspended membrane, as depicted in the schematic of Fig. 1a. Here the sputtered Mo thickness was ~100 nm and the method used for the deposition is discussed in more detail in the “Methods” Section. The actual process used for transferring the MoS_2_ from the bulk crystal to the SiO_2_/Si substrate is adapted from the *“all-dry”* viscoelastic stamping technique first reported by Castellanos-Gomez *et al*.^33^ using a polydimethylsiloxane (PDMS) film (GelFilm by GelPak). Our adapted process using a mask aligner (described in more detail in the “Methods” section) leads to ultra-clean interfaces which we have validated via Raman spectroscopy, where even thermal annealing was not necessary to reduce *R*_*c*_, unlike prior reports^34,35^. The Raman spectroscopy and AFM analysis are shown in the Supplementary Information Section where Figure S2 validates the effectiveness of our ultra-clean transfer process used to fabricate our devices due to the absence of any residual PDMS signatures.

### Electronic Transport and Schottky-Barrier Height Determination

**Figure 2:**
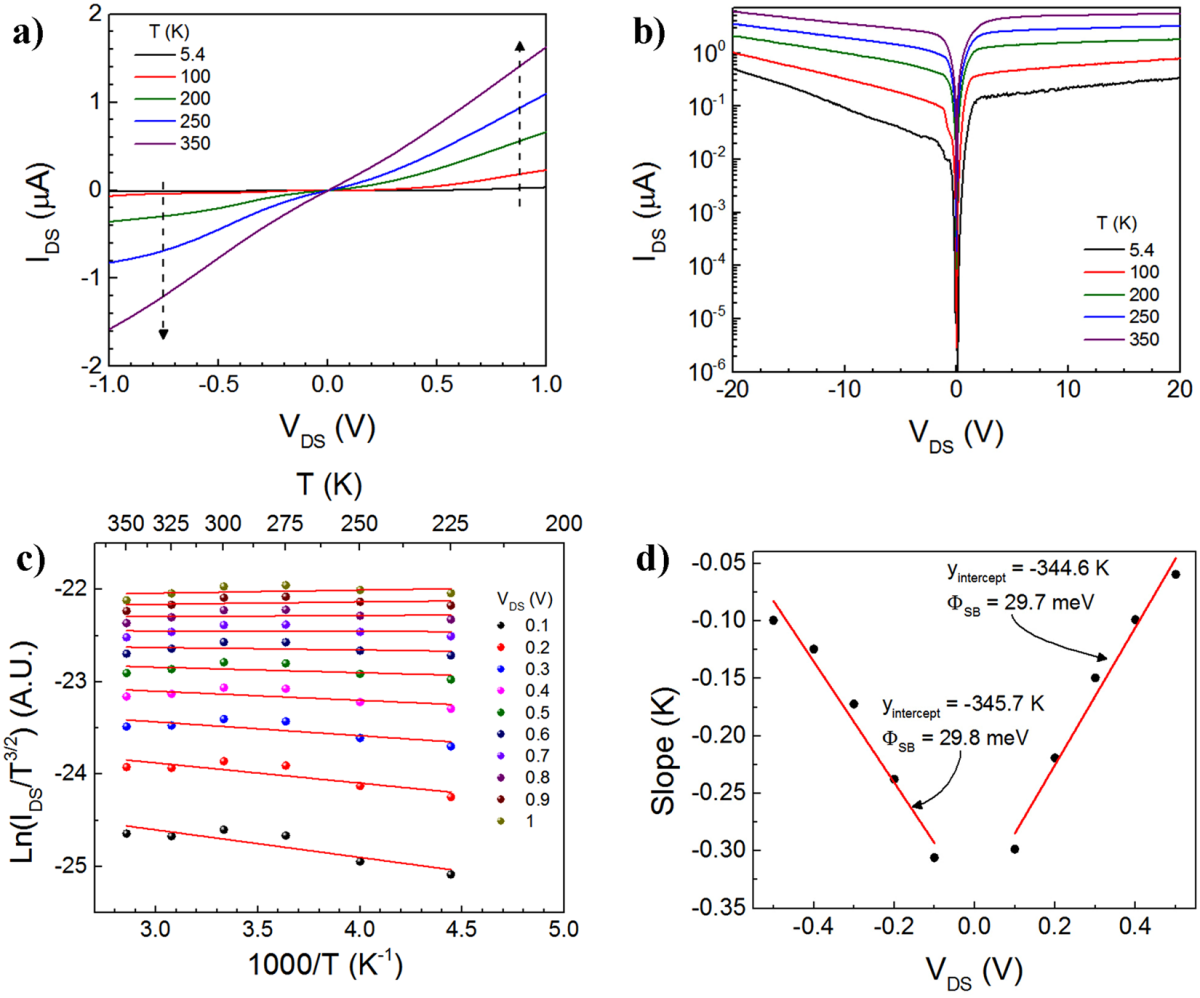
Temperature-dependent electronic response of the MSM MoS_2_ PD. *I*_*DS*_*-V*_*DS*_ Characteristic (**a**) at varying levels of temperature and (**b**) in a logarithmic scale. (**c**) Arrhenius plot at various bias voltages. (**d**) Slopes of plot 2(**c**) as a function of *V*_*DS*_. Within the linear regime at lower voltages, the *y*-intercept was used to calculate the Schottky barrier *φ*_*SB*_ ~29.8 meV in both the forward-bias and the reverse bias. The fact that the data and calculations for *φ*_*SB*_ are nearly identical in both the forward-bias and the reverse-bias regimes bodes well for the symmetric nature of the contacts.

In order to compute the Schottky-barrier height, electronic transport measurements of the devices were conducted in a vacuum probe stage (pressures ~10^−6^ Torr) at cryogenic temperatures *T* where *T* was controlled from ~5.4 K to 350 K using a closed-cycle He refrigerator. The *I*_*DS*_*-V*_*DS*_ Characteristic from drain-to-source is shown in Fig. 2a at various *T*, where measurements are conducted in the dark (dark current), and the electrodes for charge transport are those designated as in Fig. 1c (inset). As *T* increases, the *I*_*DS*_ also increases due to thermal excitation of the carriers over the Schottky barrier *φ*_*SB*_ resulting in a more Ohmic behavior at 350 K compared to, for example, 5.4 K^36^. These two-terminal measurements allow us to compute the *φ*_*SB*_. The small asymmetry in the reverse and forward bias is probably due to the difference in the contact area at the source and drain electrodes. The Fig. 2b shows the semi-log plot of *I*_*DS*_ versus *V*_*DS*_.

On the other hand, *φ*_*SB*_ is defined using the Schottky-Mott model as shown in Equation 1,

1 $${\phi }_{{SB}}\approx {{\rm{\phi }}}_{M}\,-\,{\rm{\chi }}$$

where *χ* is the electron affinity of the semiconductor (${\chi }_{{{\rm{MoS}}}_{2}}$≈ 4 eV)^37^ and *φ*_*M*_ is the metal work function (${\phi }_{{\rm{Mo}}}$ ≈ 4.3 eV)^38^. Therefore, the calculated *φ*_*SB*_ is ~0.3 eV. However, a variation can result from the alteration of *χ* by ionization energy due to surface contaminants^39^. As stated previously, a low *φ*_*SB*_ is desirable in order to improve the PD device efficiency so photocarriers are collected more efficiently at the electrodes. As the temperature increases, the *I*_*DS*_ increases with *T*, since the carriers have a higher chance of surmounting the energy barrier at the interface given their higher thermal energy. From the *I*_*DS*_*-V*_*DS*_ data collected as a function of *T*, the activation energy ${E}_{a}=({\phi }_{{SB}}-\frac{{V}_{{DS}}}{n})$ can be calculated from the slopes in the Arrhenius plot, i.e. *ln(I*_*DS*_*/T*^*3/2*^) versus *1000/T* at different *V*_*DS*_ biases is shown in Fig. 2c, from the modified thermionic emission model for a 2D material system^40^.

2 $${I}_{{DS}}={\rm{A}}{A}^{\ast \ast }{T}^{3/2}{\exp }[-\frac{q}{{k}_{B}T}({\phi }_{{SB}}-\frac{{V}_{{DS}}}{n})]$$

Here, *A* is the contact area of the junction, A** is the two-dimensional Richardson constant, *k*_*B*_ is the Boltzmann constant (~8.617 × 10^−5^ eV/K), and *q* is the electron charge (~1.602 × 10^−9^ C). Furthermore, the calculated slopes of the Arrhenius plot or low-bias voltages up to 0.5 V, are subsequently plotted at various *V*_*DS*_ levels (see Fig. 2d). From this, the Schottky barrier height of the Mo-MoS_2_ interface was extracted using Equation 3 below,

3 $${y}_{{intercept}}=-\frac{q}{{1000}{k}_{B}}{\phi }_{{SB}}$$

From the Arrhenius plot in the high temperature regime above 200 K, the temperature dependency of the mobility is dominated by the electron-phonon scattering, where the 2D thermionic emission model is applicable^41^. From this data, the Schottky barrier was empirically computed to be *φ*_*SB*_ = 29.8 meV and 29.7 meV in reverse and forward bias regimes, respectively, demonstrating the fairly symmetric nature of our contacts, and the low magnitude of the barrier heights for the MoS_2_–Mo interface; this is amongst the lowest empirically obtained *φ*_*SB*_ values to date, as compared to previous reports for ML MoS_2_ shown in Table 1 after benchmarking. In addition, the sign of the Schottky barrier confirms the *n*-type character of our MoS_2_, consistent with prior reports^8^.

**Table 1:** Comparison of *φ*_*SB*_ from prior reports for multilayer MoS_2_ (two or more layers) using different metal contacts in the absence of an applied gate voltage.

Electrodes	*φ*_*SB*_ (meV)	Ref.
Py	−5.7	^30^
Mo	29.8	This work
Sc	30	^29^
Ti	65	^28^
Au	126	^27^
Ni	150	^29^
Pt	230	^29^
Pd	400	^27^

### Optoelectronic Response

**Figure 3:**
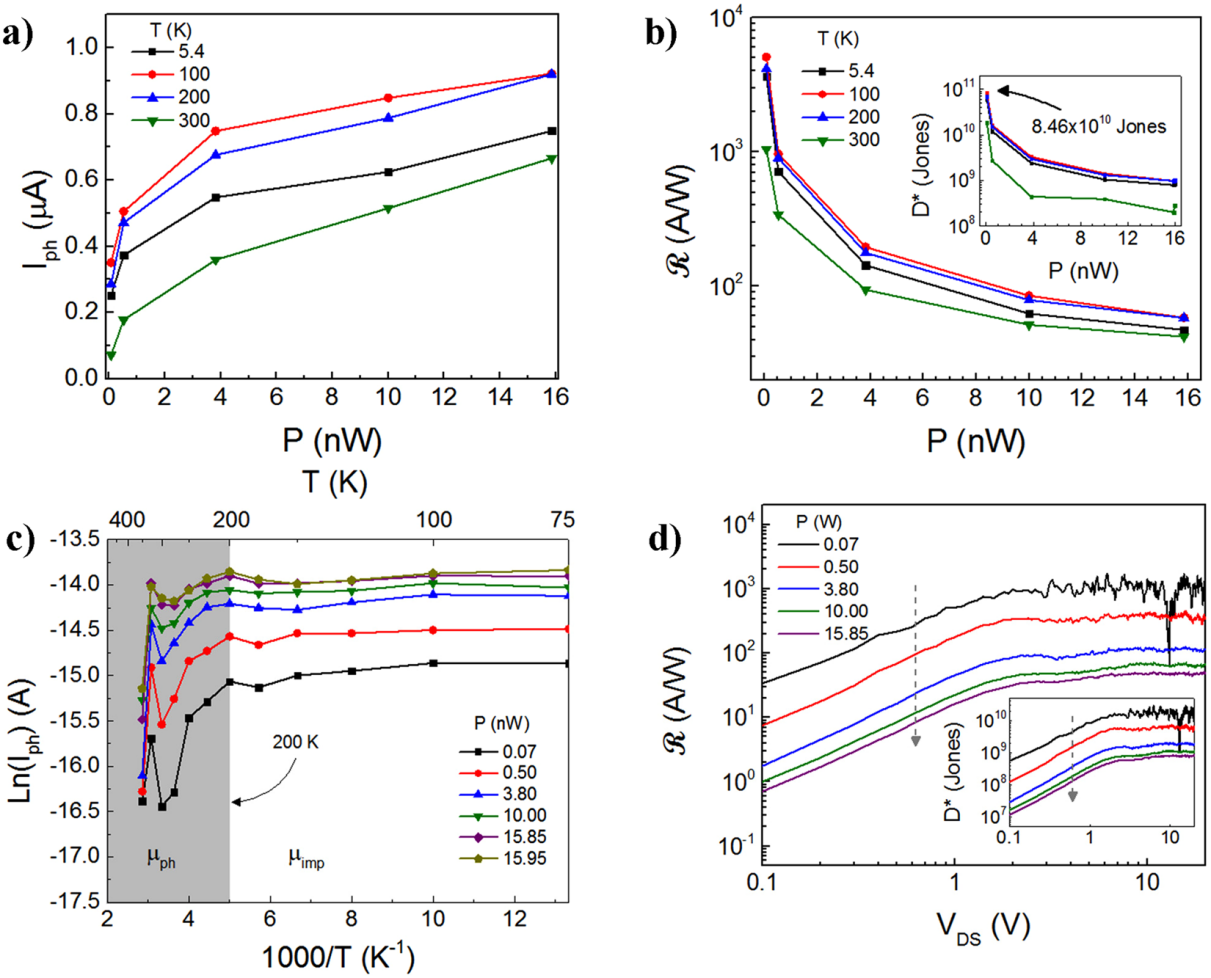
Optoelectronic response of the PD measured at a forward-bias of 5 V. (**a**) *I*_*ph*_ and (**b**) ${\mathcal R}$ and D* (inset) as a function of incident optical power *P*. The highest photosensitivity is exhibited at 100 K. (**c**) The *I* is directly proportional to the total carrier mobility *μ* where 1/*μ* *=* *1/μ*_*ph*_ + *1/μ*_*imp*_. Above 200 K (shaded region) *μ* is dominated by electron-phonon scattering *μ*_*ph*_, (*μ*_*imp*_> *μ*_*ph*_), while below this temperature, *μ* is largely determined by impurity scattering *μ*_*imp*_ (*μ*_*ph*_*> μ*_*imp*_). (**d**) ${\mathcal R}$ in the forward-bias regime at 300 K. The inset shows D* versus *V*_*DS*_, which also shows that saturation is reached for *V*_*DS*_> 2.25 V.

Next, the device was exposed to broadband white light in order to measure its photoresponse in the visible spectrum. Figure S1 in the Supplementary Information Section shows the spectrum of the white light source used. The optoelectronic measurements were conducted in vacuum and the *I*_*ph*_, ${\mathcal R}$, and D* were measured in order to quantify the PD performance. The *I*_*ph*_ is defined as the difference between the source-drain current with light exposure, *I*_*light*_, and the dark current *I*_*dark*_, i.e. *I*_*ph*_ = *I*_*light*_ − *I*_*dark*_. The ${\mathcal R}$ is defined as ${\boldsymbol{R}}={I}_{{\rm{ph}}}/({Ax}{P}_{0})$, where *P*_*0*_ is the incoming power density of the white light source, measured in mW/cm^2^, and *A* is the PD active area. In order to measure *P*_*0*_, an optical power meter (Thorlabs PM100D) was utilized. The *I*_*ph*_ and ***R*** of the device are shown in Fig. 3a and b, respectively, for *V*_*DS*_ *=* 5 V at various temperatures. The ${\boldsymbol{R}}$ was calculated to be ~1 × 10^3^ A/W and ~42 A/W at an incident power ($P={Ax}{P}_{0}$) of ~70 pW and 15.85 nW, respectively, where the measurements were conducted at 300 K at *V*_*DS*_ = 5 V. The decrease in R exhibited with *P* is due to the loss of photoexcited carriers by recombination effects, behavior commonly observed in PDs. The ${\boldsymbol{R}}$ computed represents a 20-fold improvement over the best prior reports for monolayer and 4 × 10^4^ for ML MoS_2_ MSM devices. It is important to highlight that in this experiment, no back-gate voltage *V*_*G*_ was applied.

Moreover, another parameter obtained from the optoelectronic measurements is D*, a measure of the PD sensitivity^42^ defined below,

4 $${D}^{\ast }=\frac{R{A}^{{1}/{2}}}{{({2q}{I}_{{dark}})}^{{1}/{2}}}$$

The calculated D* is ~1.74 × 10^10^ Jones at 300 K, in vacuum, for *P* ~ 70 pW and *V*_*DS*_ ≥ 2.25 V. The D* was measured as a function of *P* and the data are shown in the inset of Fig. 3b, where the maximum D* ≈ 8.46 × 10^10^ Jones at 100 K. In contrast at 300 K, D* ≈ 2.85 × 10^10^ (inset of Fig. 2b), which is an order of magnitude lower.

Figure 3c depicts the temperature dependency of *I*_*ph*_ where *I*_*ph*_ decreases for *T* > 200 K. This decay is consistent with the dominant carrier mobility scattering where the Arrhenius plot of the *I*_*ph*_ at increasing levels of *P* is shown in Fig. 3c. Below 200 K, the scattering is dominated by charge impurity scattering (gray shaded region in Fig. 3c) where the mobility due to impurity scattering *μ*_*imp*_ is limiting the transport. Above this temperature, the mobility is determined largely by electron-phonon scattering *μ*_*ph*_^43^. Additionally, ${\boldsymbol{R}}$ is shown as a function of *V*_*DS*_ in Fig. 3d, where a saturation is seen for *V*_*DS*_ > 2.25 V, while the inset similarly shows the D* dependence.

### Three-terminal Gating Measurements

**Figure 4:**
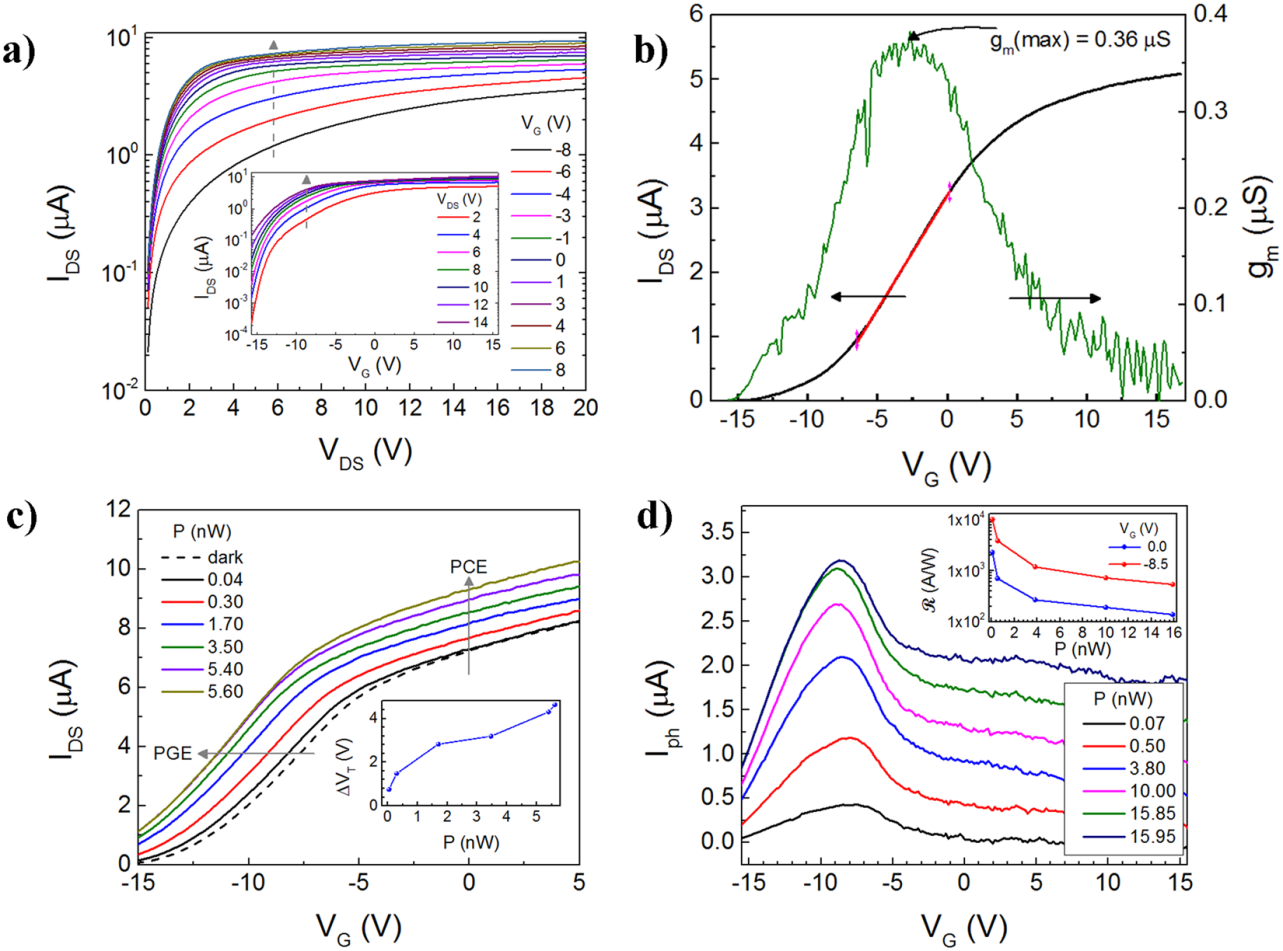
Three-terminal optoelectronic measurements in the MSM suspended MoS_2_ PD. (**a**) *I*_*DS*_*-V*_*DS*_ characteristic at varying *V*_*G*_ in dark. Inset shows *I*_*DS*_*-V*_*G*_ characteristics from which an ON/OFF ratio was calculated to be ~10^4^ at *V*_*DS*_ = 2 V. (**b**) *I*_*DS*_*-V*_*G*_ transfer characteristics for varying levels of *P* at *V*_*DS*_ = 2 V, from which a maximum *μ*_FE_ of ~42.7 cm^2^V^−1^s^−1^ was calculated at peak *g*_*m*_ ~ 0.36 μS based on Equation 5. (**c**) *I*_*ph*_ as a function of *V*_*G*_ to analyze the photocurrent generation mechanism, where the highest ${\mathcal R}$was found at ~−8.5 V. The photogating effect (PGE) is also seen to be effective here since a shift in voltage of ~−1.1 V is seen and the shift to the left indicates p-type trap states. At the same time, the photoconductive effect (PCE) appears to be the dominant photocurrent generation mechanism since *I*_*ph*_ shifts up in the y direction^45^. (**d**) *I*_*ph*_ as a function of *V*_*G*_, the highest sensitivity is shown at *V*_*G*_ = −8.5 V where the conductance is the minimum. The inset shows ${\mathcal R}$ as a function of *P* at *V*_*G*_ = 0 V and −8.5 V, respectively for *V*_*DS*_ = 5 V. All the measurements where performed at 300 K and in vacuum.

Three-terminal measurements were conducted at 300 K to obtain the ON/OFF ratio, field effect mobility *μ*_FE_, transconductance *g*_*m*_, and the optical response was measured as a function of *V*_*G*_ where *V*_*G*_ is the back-gate voltage shown in the schematic of Fig. 1a. The *V*_*G*_ was applied to the probe stage chuck upon which the ~100 Ω-cm Si substrate was mounted. From the characteristic curves *I*_*DS*_*-V*_*DS*_ at various *V*_*G*_ as shown by the data in Fig. 4a, the ON/OFF ratio was calculated to be ~10^4^ from the *I*_*DS*_ ratio between the ON state (*V*_*G*_ = 15.5 V) and the OFF state (*V*_*G*_ *=* −15.5 V). Afterwards, the *μ*_FE_ was calculated from the linear region in the *I*_*DS*_*-V*_*G*_ transfer characteristic (Fig. 4b) using the Equation 5 below,

5 $${\mu }_{{FE}}=\frac{L}{W}\frac{{g}_{m}}{{C}_{t}{V}_{{DS}}}$$

where *g*_*m*_ *=* *dI*_*DS*_*/dV*_*G*_ and *C*_*t*_ *=* 52.3 μF/m^2^ is the total capacitance; here ${C}_{t}={C}_{{\rm{ox}}}{C}_{{\rm{air}}}/({C}_{{\rm{ox}}}+{C}_{{\rm{air}}})$ which is the series capacitance of the oxide layer ${C}_{{\rm{ox}}}={\varepsilon }_{0}{\varepsilon }_{{\rm{ox}}}/{t}_{{\rm{ox}}}$ and the capacitance of air ${C}_{{\rm{air}}}={\varepsilon }_{0}{\varepsilon }_{air}/{t}_{air}$. Here the oxide thickness *t*_*ox*_ ~ 270 nm and the relative permittivity of the oxide *ε*_*ox*_ = 3.6 was used. The air gap was assumed to be equal to the Mo layer thickness *t*_*air*_ ~ 100 nm. The *μ*_FE_ was calculated at different *V*_*DS*_, where the maximum *μ*_FE_ ~ 42.7 cm^2^V^−1^s^−1^ at a peak *g*_*m*_ ~ 0.36 μS at *V*_*DS*_ = 2 V (Fig. 4b). This is in agreement with previous reports on ML MoS_2_ where *μ*_*FE*_ varies from 21 cm^2^V^−1^s^−1^ to 184 cm^2^V^−1^s^−1^ ^17,29,44^.

Furthermore, the photoresponse was analyzed as a function of *V*_*G*_ as shown by the data in Fig. 4c. From the *I*_*DS*_*-V*_*G*_ plot, it can be inferred that the photocurrent generation mechanism in the MoS_2_ MSM PD appears to be due to the photoconductive effect (PCE), since the photocurrent increases in the positive y direction as the power increases, for all power levels tested^45^. At the same time, the PD also appears to have a small contribution from the photogating effect (PGE) that can be readily validated by the threshold voltage *V*_*T*_ shift (calculated from the *g*_*m*_ extrapolation of the linear region) in which *ΔV*_*T*_ *=* 0.74 V and increases up to 4.6 V when the incident power increased from 40 pW to 5.6 nW, as shown in the inset of Fig. 4c. Other reports on bilayer MoS_2_ demonstrate a *V*_*T*_ shift of 18 V in vacuum^46^. The shift to the left indicates that the trap carriers have a *p*-type character, which appears to be in agreement with our material, where hole-trap states are likely to be present due to Sulphur vacancies in the MoS_2_^45^. Moreover, a comparison of the photoresponse of our device with and without the application of *V*_*G*_ *i*s shown in Fig. 4d at varying levels of *P* ranging from 70 pW up to 15.95 nW. At *P* ~ 70 pW a maximum in *I*_*ph*_ occurred when *V*_*G*_ ~ −8.5 V, where the corresponding ${\boldsymbol{R}}$ ≈ 1 × 10^4^ A/W in contrast to ${\boldsymbol{R}}$ ≈ 2.3 × 10^3^ A/W for *V*_*G*_ ~ 0 V, confirmed by the data in the inset of Fig. 4d and the conductance is minimum.

### Dynamics of Photogenerated Carriers

Here the photogenerated carrier dynamics of our MoS_2_ MSM detector is analyzed in greater detail. The photocurrent generation mechanism is explained using the power law relationship between *I*_*ph*_ and the incident light power ${I}_{{ph}}\propto {P}^{\gamma }$ where the exponential *γ* depends on the current mechanism that is dominant.

**Figure 5:**
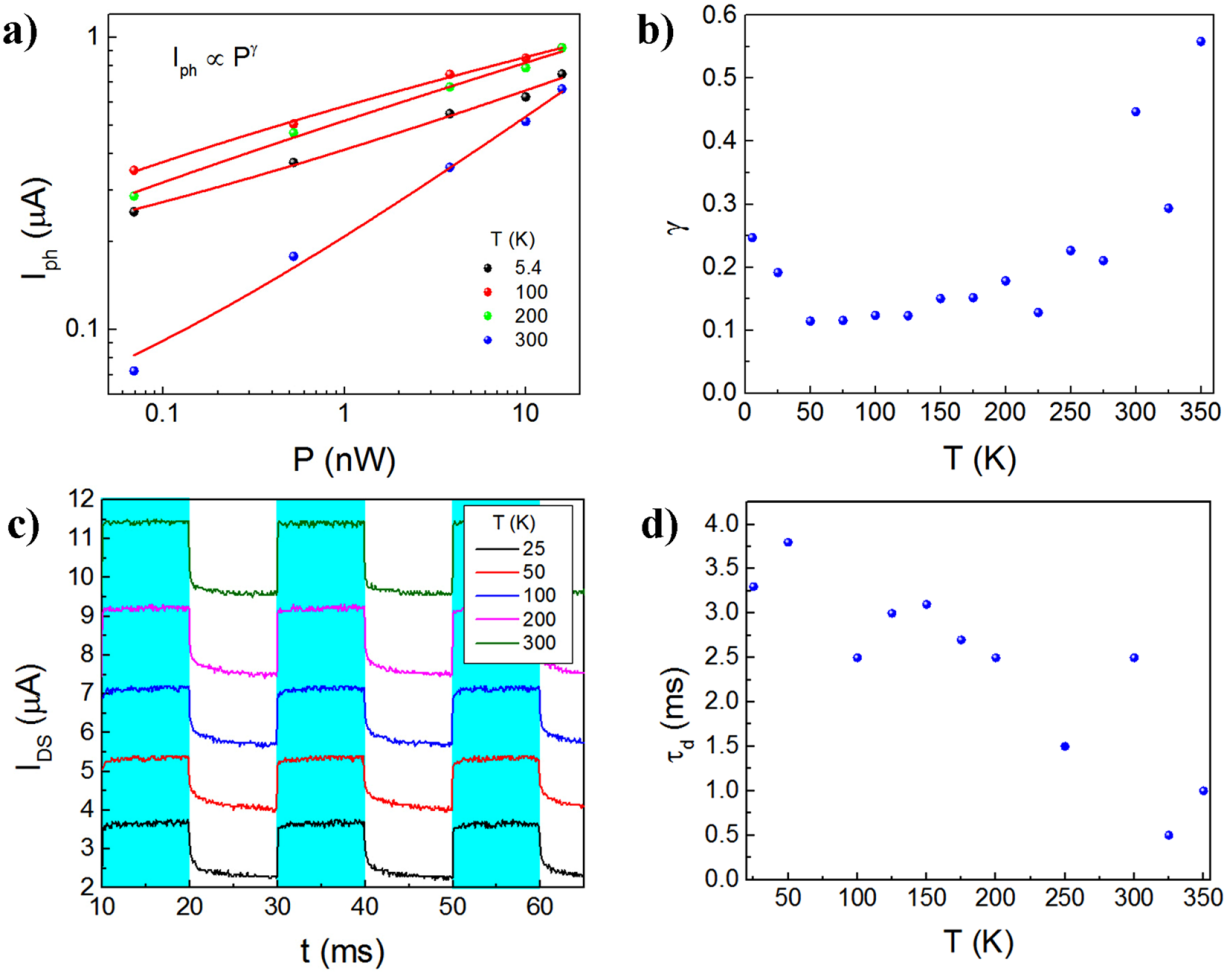
Dynamics of photogenerated carriers at 300 K, *V*_*DS*_ = 5 V where the measurements are done in vacuum. (**a**) *I*_*ph*_ versus *P* exhibiting a non-linear behavior. The exponent *γ* is extracted from the power law ${I}_{{ph}}\propto {P}^{\gamma }$. (**b**) The *γ* is plotted as a function of *T* and yields values for *γ* between 0.2 and 0.8, suggestive of a fast recombination time for the free carriers which is consistent with the PCE. (**c**) Time-resolved photoresponse at *T* ranging from 20 K − 350 K and *P* ~ 12.1 nW. The cyan area corresponds to the period when the incident light is ON, while the white area represents the decay response of the photocurrent with the light OFF. The *τ*_*r*_ shows a faster response than our acquisition system (<100 μs) while the *τ*_*d*_ decreases from 3.3 ms at 25 K to 0.5 ms at 325 K. (**d**) *τ*_*d*_ as a function of *T*, where *τ*_*d*_ is reduced as *T* increases.

In most prior reports on MoS_2_ PDs, *I*_*ph*_ exhibits a near-linear dependency with the incident power. However, some manuscripts report a non-linear dependency with values 0.5 ≤ *γ* *≤* 0.7^47^. Typical *γ* values in simple crystals correspond to *γ* *=* 0.5 in bimolecular processes while *γ* *=* 1 is operative in monomolecular processes. Moreover, in disordered semiconductors *γ* ≈ 2/3 which is common for scenarios where a large number of trap states are present^48^. In addition to mid-gap trap states, the non-linear dependency can be caused by other effects, such as the photothermoelectric effect (PTE) and the photovoltaic effect (PVE)^49^. For the PTE, a temperature gradient due to photons is required, but this can be ruled out for the devices here since the entire active area of the MoS_2_ was exposed to the incoming radiation. Moreover, the photocurrent at zero bias is on the order of tens of pA so that the PVE contribution, in which an internal electric field results in charge separation of the *e-h* pairs, is minimum or null. The results of our photocurrent and power dependency are shown in Fig. 5a and b, where *γ* varies from 0.11 to 0.56. Therefore, based on this analysis, the PCE is indeed the dominant mechanism in the devices reported here, and is suggestive of mid-gap trap states involved in the PD device operation.

In order to shed insights on the photocurrent generation dynamics, the time-resolved photoresponse was analyzed by determining the rise time *τ*_*r*_ and the decay time *τ*_*d*_ of *I*_*ph*_*;* the instrumentation setup for this analysis is presented in more detail in the Supplementary Information Section, Figure S3. From prior reports, a fast photocurrent decay is related to the recombination of free carriers while a slow decay is an indication of thermal de-trapping of carriers, for example through the channel when the semiconductor is in direct contact with the substrate. This last effect causes persistent photoconductivity (PPC), commonly observed in monolayer MoS_2_^50^. Our time-resolved measurements comprise of a broadband light source (*P* *=* 12.1 nW) where the pulses of radiation have a duration of 10 ms and the device is biased at *V*_*DS*_ *=* 5 V (see Fig. 5c). The *τ*_*r*_ of our device is faster than the instrumentation acquisition time, in the Keysight B1500A, where a maximum resolution of 100 μs is evident from *T* *=* 5.4 K to 350 K in Fig. 5c. Conversely, the *τ*_*d*_ is reduced from 3.3 ms at 25 K to 500 μs at 325 K (see Fig. 5d), in contrast to previous reported values where *τ*_*d*_ as high as 500 s and 200 s have been reported^20,51^. As a result, the time-resolved analysis of the photocurrent is consistent with the PCE that appears operative in our devices, which is an effect that is amplified by mid-gap trap states (e.g. hole-trap states or other defects such as interstitials) in the MoS_2_ diaphragm, which provide additional internal gain mechanisms. Therefore, we conclude that the suspended architecture of our MSM PD enables a fast photoresponse and significantly improves the PD performance metrics.

### Spectral Response of PD

**Figure 6:**
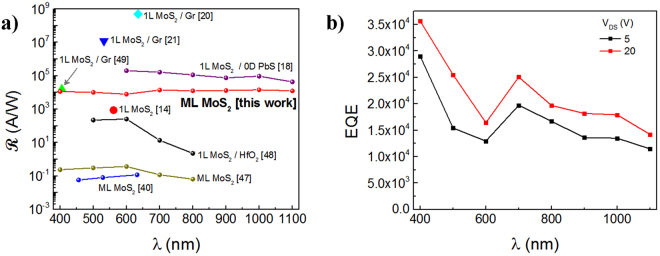
Spectral variation of the Photoresponse at *P* (average) ~14.5 pW. (**a**) A graphical illustration of the comparison of R values with prior work. As noted, the R reported here exhibits high values over the entire spectral regime from 400–1100 nm, ranging from ~8 × 10^3^ A/W to ~1.4 × 10^4^ A/W, which is a significant improvement over prior work on ML and 1 L MoS_2_. (**b**) The EQE was determined to vary from 3.6 × 10^4^ to 1.4 × 10^4^ within the 400 nm–1100 nm range, respectively.

In order to demonstrate the ultra-high broadband photoresponse of the MoS_2_ MSM PD, a tunable laser source with 1 nm of resolution, was used to measure the photoresponse as a function of wavelength from 400 nm to 1100 nm (spectral range of the Fianium LLFT Contrast NKT Photonics tunable laser source) in 100 nm increments. The measurements were conducted in vacuum and from Fig. 6a, it can be seen that ${\boldsymbol{R}}$ varied from ~8 × 10^3^ A/W to ~1.4 × 10^4^ A/W over the 400 nm to 1100 nm spectral range at *V*_*DS*_ ~ 20 V. Thus, here we clearly demonstrate the broadband nature of our PD which exhibits an outstanding ${\boldsymbol{R}}$ over the entire spectral regime from the UV to the near-IR. The PD reported here is more than 10^4^ times superior compared to other ML MoS_2_ PDs and is 20 times more sensitive than its 1 L counterparts. On the other hand, as mentioned previously in the “MoS_2_ Device Architecture” Section, the implementation of more complex systems, such as quantum dots and MoS_2_/graphene heterostructures improves the device performance^26^.

From the ${\mathcal R}$ calculation, the external quantum efficiency (EQE) was determined as follows,

6 $${EQE}=R\frac{{hc}}{{\rm{\lambda }}{q}}$$

where *h* is Plank’s constant (~4.135 × 10^−15^ eV·s), *c* is the speed of light (~3 × 10^8^ m/s),and *λ* is the wavelength of the incoming light. The EQE is shown in Fig. 6b as a function of *λ*, where EQE varies from 8 × 10^3^ to ~1.4 × 10^4^ within the 400 nm–1100 nm range. In contrast with photovoltaic cells, when *V*_*DS*_ is applied in PDs, the EQE can be higher than 1 due to the external electric field contributing to extend the lifetime of the photogenerated carriers so more than one *e-h* pair is generated per incident photon. The peak observed at 700 nm coincides with the direct hot-luminescence *A1* peak at 1.8 eV. On the other hand, the decay observed in Fig. 6b at both levels of *V*_*DS*_ (i.e. 5 V and 20 V) as λ increases is due to the incoming radiation approaching the cutoff wavelength corresponding to the MoS_2_ indirect bandgap, generally at ~1.2 eV^52–54^.

## Methods

The metal contacts were fabricated from a photolithography mask designed to allow the suspension of the MoS_2_ on top of the contacts. Firstly, a 270 nm SiO_2_/Si substrate was used for the transfer of MoS_2_ to yield a high optical contrast between the substrate and the MoS_2_ nanomembrane. The substrate was cleaned using piranha to reduce surface contaminants prior to the transfer. Secondly, standard photolithography was used with AZ 5214E-IR photoresist to define the contact regions, after which point 100 nm of sputtered Mo was deposited at 200 Watts and 3 mTorr Ar pressure. After deposition, the Mo was lifted-off with acetone and the surface was further cleaned using stripper (AZ Kwik Strip Remover). The MoS_2_ nanomembrane was then mechanically exfoliated from the bulk crystal (2D Semiconductor Inc.) using low-tac blue tape (Semiconductor Equipment Corp.) and aligned onto the electrodes on the Si substrate, adapted from the viscoelastic stamping process reported previously^33^. Here a Karl Suss MJB-3 mask aligner was used for the alignment and subsequent transfer. The SiO_2_/Si substrate with the Mo electrodes was held on the wafer chuck. Meanwhile, the MoS_2_ from the blue tape was then attached to the PDMS Gel-Film which adhered to a clear 4-inch x 4-inch glass plate mounted onto the mask aligner. The MoS_2_ nanomembrane on the Gel-Film-glass plate assembly was then aligned to the Mo-electrodes on the Si substrate using the mask aligner. The opto-electronic measurements were conducted using a state-of-the-art Lakeshore CRX-4K Cryogenic Probe Stage and an ultra-low noise Semiconductor Parameter Analyzer (Keysight B1500A). The contacts were electrically cycled up to ~2 μA to drive-off potential interfacial contaminants between the Mo-contact and the MoS_2_ nanomembrane; no thermal annealing was used in our device fabrication process. In addition, the device was kept in vacuum (~10^−6^ Torr) for a duration of two weeks to further remove absorbed interfacial residues and moisture. The broadband white light source utilized in our optoelectronic measurements was a Thorlabs LED model SOLIS-1C driven by a Thorlabs DC2200. The tunable spectral measurements were conducted using a tunable laser source, the Fianium LLFT Contrast from NKT Photonics. Both the broadband and narrow-band sources were calibrated using the Thorlabs optical power meter PM100D. The laser excitation wavelength used in the Raman and photoluminescence measurements was 532 nm where the output laser power was ~33.5 mW. The atomic force microscopy measurements were performed using the Bruker Multimode-8 Microscope at ambient conditions in tapping mode.

## Conclusions

In this work, Mo has been utilized as the bottom metal contact for our mechanically exfoliated ML mesoscopic MoS_2_ PD. This metal has been previously reported via theoretical simulations as an alternative for contacting MoS_2,_ and here we indeed empirically demonstrate, for the first time, the ultra-high performance obtained with Mo contacted MoS_2_ to yield a low Schottky barrier at the interface. Photoluminescence (PL) measurements were conducted in the supported and suspended regions of the MoS_2_ membrane, to validate that our devices are indeed composed of suspended MoS_2_ nanomembranes. In addition, the electronic and opto-electronic properties were characterized as a function of temperature and important device parameters were computed, which included *φ*_*SB*_ ~ 29.8 meV, ${\boldsymbol{R}}$ and D* ~ 10^4^ A/W and 10^11^ Jones, respectively, for *V*_*DS*_ > *2*.*25* *V and P* = *14*.*5* *pW* at 300 K in vacuum over a wide spectral regime from 400 nm to 1100 nm. The response time measured was fast where *τ*_*r*_ < 100 μs and *τ*_*d*_ ~ 2.5 ms. The 4 × 10^4^ times higher ${\boldsymbol{R}}$ compared to previous ML MoS_2_ reports is likely to arise from a combination of factors. This includes the use of an ultra-clean and all-dry MoS_2_ nanomembrane visco-elastic transfer method, the suspended nature of the MoS_2_ device architecture which reduces the electron-phonon and impurity scattering mechanisms at the interface, the use of Mo as our low *φ*_*SB*_ contact metal, and the ML mesoscopic nature of our MoS_2_ nanomembrane that expands the detection spectra. To our knowledge, our results represent the best PD performance metrics obtained to date for any ML MoS_2_ PDs based on a MSM architecture, excluding heterostructures, and also in fact exceeds the performance of 1 L MoS_2_ devices. Thus, we confirm the potential of this material for photocurrent generation not only in its monolayer form shown extensively in previous reports, but also in its ML, suspended mesoscopic form, as we demonstrate here.

## Electronic supplementary material

Supplementary Information
