# Supplementary material for: Ultra-high Photoresponsivity in Suspended Metal-Semiconductor-Metal Mesoscopic Multilayer MoS2 Broadband Detector from UV-to-IR with Low Schottky Barrier Contacts
Source: Sci Rep. 2018 Jan 19;8:1276. doi: 10.1038/s41598-018-19367-1 (PMC5775306; doi:10.1038/s41598-018-19367-1)
Supplement: Supplementary file 1 — Supplementary Information [file 41598_2018_19367_MOESM1_ESM.pdf]

Supplementary Information

Ultra-high Photoresponsivity in Suspended Metal-Semiconductor-Metal Mesoscopic Multilayer MoS_2 Broadband Detector from UV to IR with low Schottky Barrier Contacts

Gustavo A. Saenz[1,2,3], Goran Karapetrov[4], James Curtis[4], and Anupama B. Kaul[1,2,3]*

[1]Department of Materials Science and Engineering, PACCAR Technology Institute, The University of North Texas, Denton, TX 76203

[2]Department of Electrical Engineering, The University of North Texas, Denton, TX 76203

[3]Department of Electrical and Computer Engineering, The University of Texas at El Paso, El Paso, TX 79968

[4]Department of Physics, Drexel University, Philadelphia, PA 19104

*E-mail: anupama.kaul@unt.edu

Spectrum of the Broadband Light Source

The broadband LED light source spectra is shown in Figure S1. This source was utilized for the optoelectronic measurements, including the time-resolved measurements for the data shown in Figures 3, 4, and 5 of the main manuscript.

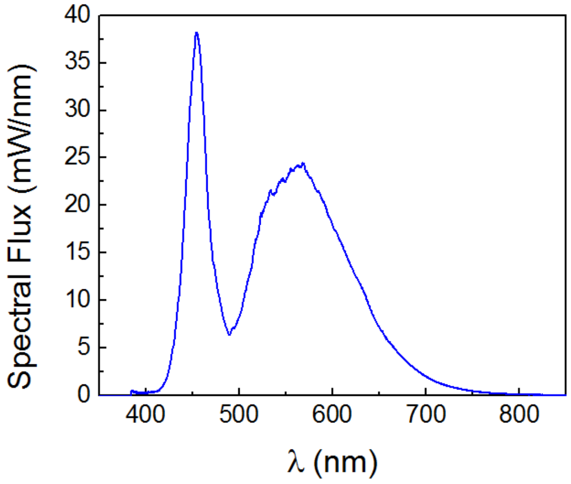

Figure S1. Broadband light source spectra. Reproduced from Thorlabs specification sheet for the LED model SOLIS-1C source.

The MoS_2 Ultra-clean Transfer Process

The actual process used for transferring the MoS_2 from the bulk crystal to the SiO_2/Si substrate is adapted from the "*all-dry*" viscoelastic stamping technique first reported by Castellanos-Gomez *et al.*[1] using a polydimethylsiloxane (PDMS) film (GelFilm by GelPak). Our adapted process with a mask aligner (described in more detail in the "Methods" section) leads to ultra-clean interfaces which we have validated via Raman spectroscopy, where even thermal annealing was not necessary to reduce R_c, unlike prior reports.[2,3] The optical image of the fabricated structure is shown in Figure S2a, where the drain (*D*) and source (*S*) electrodes are illustrated for charge collection. The AFM scan of the nanomembrane lodged on the Mo electrodes was done in tapping mode and yielded a thickness of ~ 62 nm in going from point A to point B, as shown in Figure S2b. From this data, the root-mean-squared (RMS) roughness of the MoS_2/sputtered Mo surface was determined to be ~ 1 nm. Next, Raman spectroscopy was used to validate signatures of remnant organics or PDMS contaminants that may have been generated via the transfer stamping process and can further compromise device performance. The spectrum of the polymer on the SiO_2/Si substrate was measured for the bare PDMS GelFilm used for the transfer that served as our reference (Figure S2d); here we confirm these peaks are indeed those attributed to PDMS, as shown by the spectra in red in Figure S2d. The Raman spectra obtained for an independent MoS_2 nanomembrane was then measured that was transferred using the PDMS onto SiO_2/Si to seek signatures of remnant residues, and the data are represented by the blue spectra in Figure S2d which is superimposed onto the PDMS spectra. Here we clearly see the absence of any residual PDMS since the peaks characteristic of PDMS at, for example, k = 2906 cm^{-1}, are not evident which confirms the cleanliness of our transfer process. Moreover, bulk MoS_2 has the in-plane

vibrational mode E^{1}_{2g} at 382.7 cm^{-1} and the out-of-plane A_{1g} mode at 407.6 cm^{-1},[4] as confirmed by the data in the inset of Figure S2d; the peak at 520.2 cm^{-1} in the blue spectra is attributed to the Si substrate. From the inset in Figure S2d, the Raman shift Δk between the E^{1}_{2g} and the A_{1g}, i.e. $\Delta k = E^{1}_{2g} - A_{1g}$ = 24.9 cm^{-1}, corresponding to bulk MoS_2.[4]

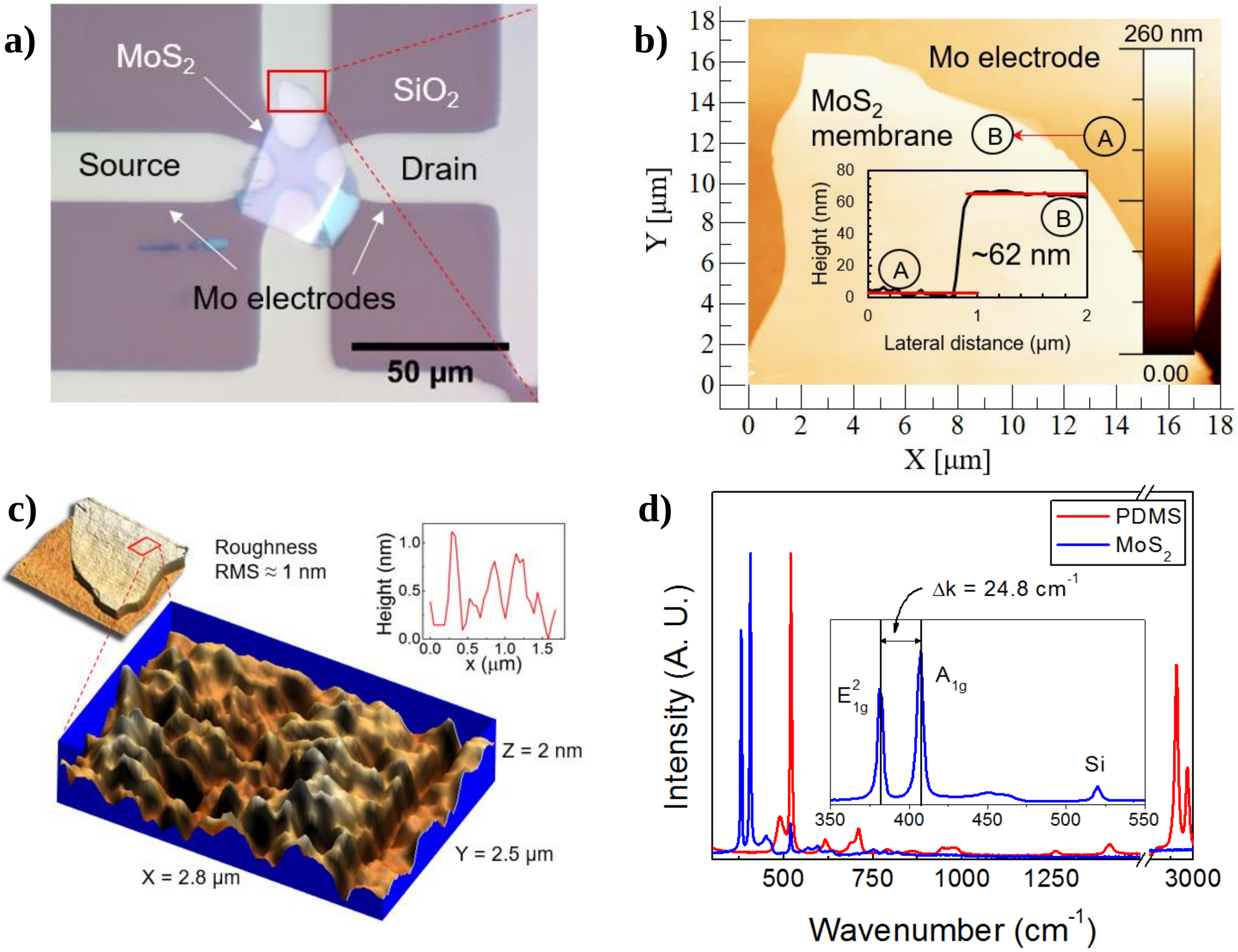

Figure S2. (a) Optical micrograph of the PD device showing the Mo bottom contacts on SiO_2/Si substrate. (b) AFM scan obtained in tapping mode as the tip traversed from point A (Mo) to point B (MoS_2), where the MoS_2 diaphragm thickness was determined to be ~ 62 nm for this particular device. (c) The RMS roughness of the MoS_2 surface was ~ 1 nm which was comparable to the sputtered Mo surface roughness, indicating minimal residues from the PDMS. (d) Normalized

Raman spectra of PDMS and MoS_2, shown in red and blue, respectively, to seek-out signatures of any residues after the PDMS stamping process. The absence of peaks at wavenumbers > 2900 cm^{-1} in the MoS_2 nanomembrane spectra confirms the absence of residual PDMS, and validates the ultra-clean nature of the stamping and device fabrication process.

Optoelectronic Measurement Setup

The optoelectronic measurements setup is shown in Figure S3, to conduct the time-resolved measurements using a pulse modulator for the broadband LED light source. A Semiconductor Parameter Analyzer B1500A was used to gather the V_{DS} - I_{DS} data using a medium power source-measurement unit (SMU), as shown in Figure S3. The device under test (DUT) is shown as it is mounted on the chuck of the cryogenic probe. The SMU utilized for the measurement has an acquisition time limit of 100 µs; however, the response time τ_r of the PD was faster than the acquisition time of the SMU, and hence could not be determined. On the other hand, the decay time τ_d was measured and the data are shown in Figure 5c and (d) of the main manuscript for measurements from 5.4 K to 350 K.

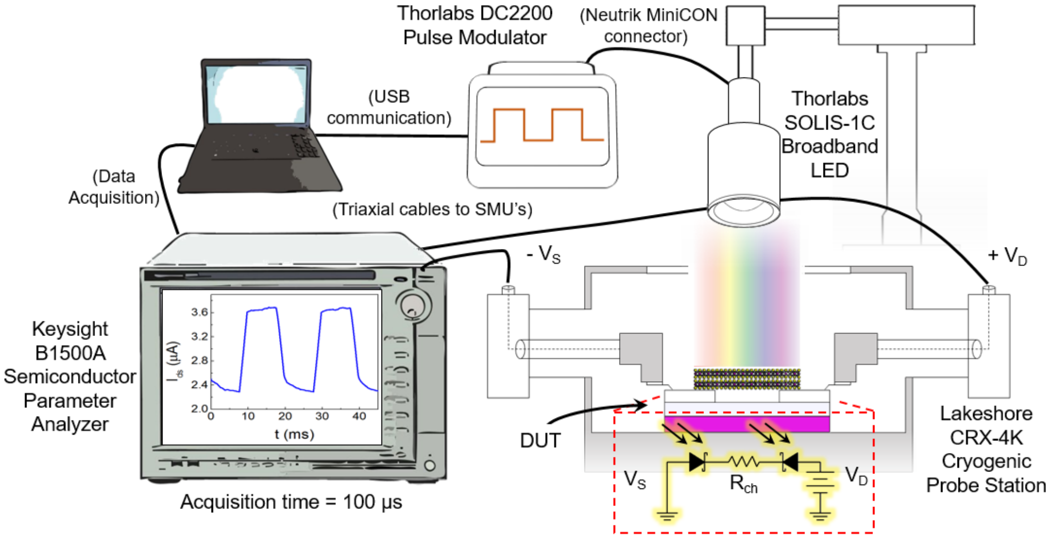

Figure S3. Time-resolved optoelectronic measurements setup using a broadband LED light source for MoS_2 MSM PD measurements. The computer served to control the frequency and power of

the broadband LED SOLIS-1C via the Thorlabs DC2200 pulse modulator and for data acquisition (I_{DS} vs t) from the B1500 Semiconductor Parameter Analyzer. In addition, the DUT equivalent circuit (highlighted in yellow) is shown for an n-type MSM PD corresponding to two Schottky diodes facing to each other on the cathode side with a channel resistance R_{ch} in between.

Conductance as a Function of V_G

Next, the conductivity was calculated as a function of the V_G (Figure S4), where the minimum conductance is obtained at V_G = -8.6 V, the voltage at which the highest photoresponse is obtained.

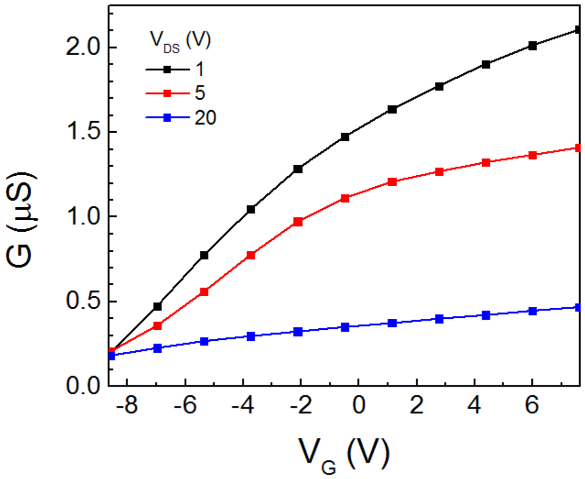

Figure S4. Conductance as a function of V_G at different applied bias.

MSM MoS_2 PDs Comparison

In this section, a comparison of the important performance parameters between two MSM mesoscopic suspended multilayer MoS_2 PDs devices, Sample 1 (S1) and Sample 2 (S2), is presented here (see Figure S5). This includes reference to the photocurrent I_{ph}, detectivity D^*, photoresponsivity $\mathscr{R}$, and normalized photocurrent-to-dark ratio (NPDR), a performance metric defined in Equation 1 below. This analysis was conducted as a function of the incident power density P_0. A performance comparison between our devices is shown in Figure S5 which shows that $\mathscr{R}$ varies from 4.7 x 10^3 A/W and 1 x 10^3 A/W for Sample 1 (S1) and Sample 2 (S2),

respectively, at V_{DS} = 5 V and 300 K. This demonstrates that our device architecture yields high performance PDs where both samples yield performance parameters that are comparable. The measurements and analysis in the main manuscript are based on S2.

$$NPDR=\mathscr{R}/I_{dark} \tag{1}$$

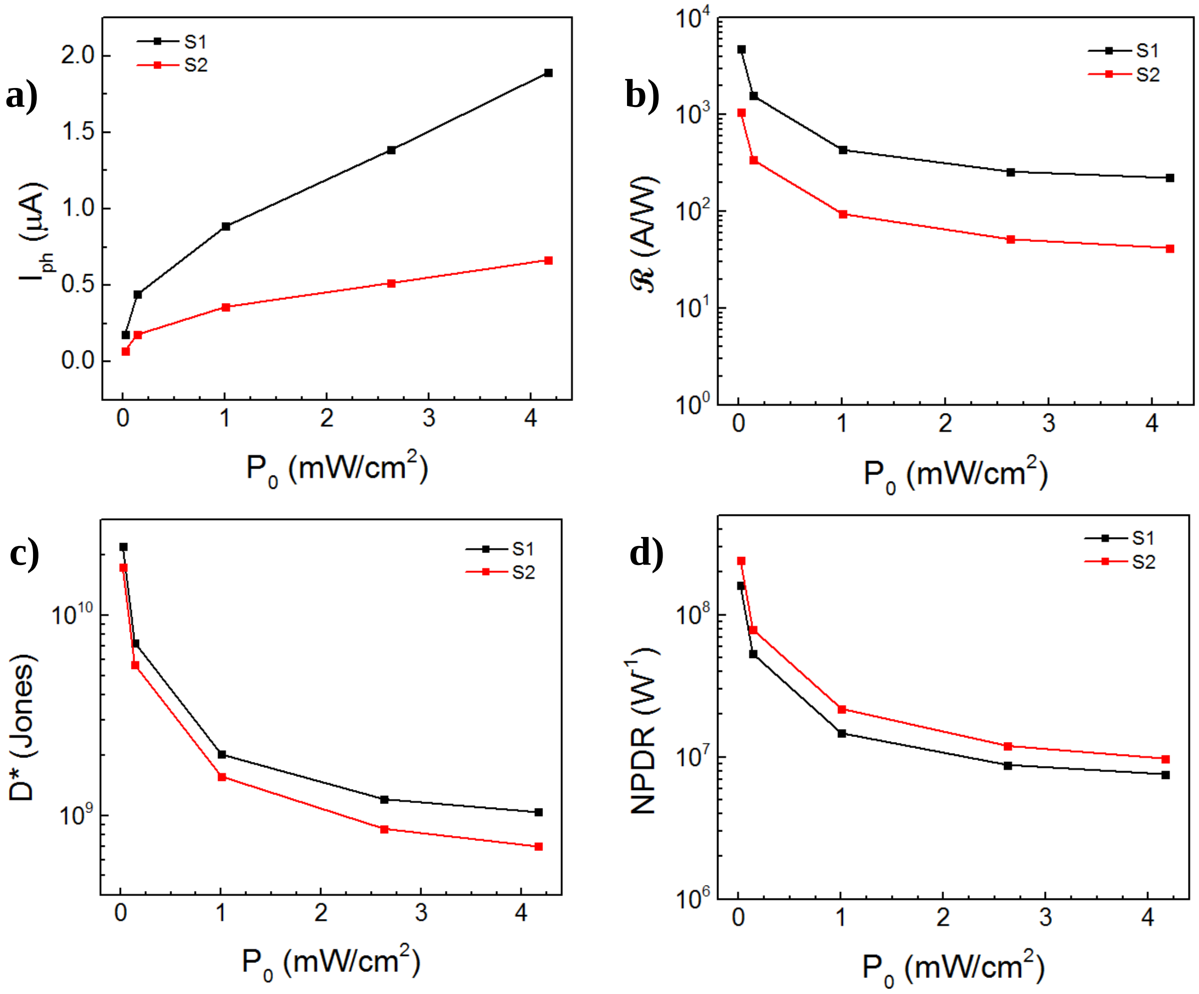

Figure S5. Comparison of performance between additional MoS_2 PDs where measurements were made at V_{DS} = 5 V and 300 K under vacuum without the application of V_G. Shown are (a) I_{ph}, (b) $\mathscr{R}$, (c) NPDR, and (c) D^* as a function of P_0, the incident power density.

References

1. Castellanos-Gomez, A. *et al.* Deterministic transfer of two-dimensional materials by all-dry viscoelastic stamping. *2D Mater.* **1,** 11002 (2014).
2. Yang, R., Zheng, X., Wang, Z., Miller, C. J. & Feng, P. X.-L. Multilayer MoS_2 transistors enabled by a facile dry-transfer technique and thermal annealing. *J. Vac. Sci. Technol. B* **32,** 61203 (2014).
3. Man, M. K. L. *et al.* Protecting the properties of monolayer MoS_2 on silicon based substrates with an atomically thin buffer. *Sci. Rep.* **6,** (2016).
4. Lee, C. *et al.* Anomalous lattice vibrations of single- and few-layer MoS_2. *ACS Nano* **4,** 2695–2700 (2010).
